# Effect of NaCl Pretreatment on the Relationship between the Color Characteristics and Taste of *Cirsium setidens* Processed Using a Micro-Oil-Sprayed Thermal Air Technique

**DOI:** 10.3390/plants12183193

**Published:** 2023-09-06

**Authors:** Yonghyun Kim, Uk Lee, Hyun Ji Eo

**Affiliations:** Special Forest Resources Division, National Institute of Forest Science, Gwonseon-gu, Suwon 16631, Republic of Korearich26@korea.kr (U.L.)

**Keywords:** *Cirsium setidens*, drying process, color characteristics, taste, health-promoting compounds

## Abstract

*Cirsium setidens* is commonly used as a food ingredient, and it is typically stored and distributed in a dried form to prolong its shelf life. In a previous study, a micro-oil-sprayed thermal air (MOTA) technique was developed, which effectively enhanced the rehydration properties and improved the color characteristics of *Cirsium setidens* after processing. Here, we investigated the relationship between the color characteristics and taste of MOTA-processed *C. setidens* and the effect of NaCl pretreatment, prior to processing, on the final quality of dried *C. setidens*. NaCl pretreatment, whether combined with the MOTA technique or not, showed improved color characteristics, in which MOTA-and NaCl+ MOTA-processed *C. setidens* manifested equal color characteristics. In contrast, NaCl + MOTA-processed *C. setidens* resulted in significantly higher values of sourness and saltiness than MOTA-processed *C. setidens* when the taste of the rehydrated *C. setidens* was examined using an electronic tongue (Astree II; Alpha MOS, Toulouse, France). Pearson correlation coefficient analysis showed that sourness and saltness were negatively correlated with Hunter a* values and positively correlated with Hunter L* and Hunter b* values, indicating that the color characteristics of dried and rehydrated *C. setidens* could be indicators of taste. Furthermore, the amounts of total phenol and chlorophyll were better preserved in *C. setidens* processed by the MOTA technique with NaCl than by the MOTA technique alone. Our data revealed that the color characteristics of dried plants are associated with the taste of processed *C. setidens*, and that the MOTA technique with NaCl pretreatment is a useful method for preserving health-promoting compounds during processing.

## 1. Introduction

*Cirsium setidens* is a wild vegetable widely cultivated and consumed as a food ingredient in South Korea. Wild vegetables are traditionally regarded as medicinal plants due to their therapeutic effects in conditions such as edema, bleeding, and hemoptysis [1]. They contain biologically active compounds, some of which are natural antioxidants [2,3,4]. Their potential pharmacological activities and functions have been reported previously [2,5,6,7]. Although *C. setidens* seems to be a valuable source of functional foods, wild vegetables rapidly lose their freshness after harvest, resulting in a short shelf life in the marketplace. Thus, most wild vegetables are dried immediately after harvest to prolong their storage and distribution.

Drying is widely used to preserve food ingredients during storage and to retain food stability by minimizing chemical and physical changes and preventing biochemical deterioration [8,9]. However, dried edible wild vegetables require a rehydration procedure to be used as food ingredients, which increases their preparation time. Moreover, insufficient rehydration methods or time lead to undesirable textural properties and color characteristics in these food ingredients [10]. Prior to drying fresh-cut fruits or edible wild vegetables, pre-drying treatments and their combinations are typically applied. Pre-drying treatments have a notable effect on the rehydration ability, color characteristics, textural properties, and flavor of dried food ingredients [8]. Recently we developed a new pre-drying method termed the “micro-oil-sprayed thermal air (MOTA) technique” [11]. The MOTA technique was based on the disruption of the cell wall by treatment with thermal air, leading to the softening of the plant tissue. Thus, each dried wild vegetable (DWV) processed using the MOTA technique showed an improved rehydration ability, coinciding with a reduction in the time to reach the maximum rehydration level. In addition, improvements in color characteristics occurred in some edible wild vegetables processed by the MOTA technique—for example, an increase in brightness and greenness. As the MOTA technique is a relatively simple and time-saving pre-drying method, it is expected to be easily combined with other pre-drying methods such as chemical pretreatment.

The electronic tongue (e-tongue), as a new technology, is an innovative method that can measure taste using metal or polymer sensors without trained panelists [12]. Various studies have been conducted using the e-tongue for its application in wines, juices, teas, and the pharmaceutical industry because it is able to examine both qualitative and quantitative determinants of taste [13,14,15,16,17,18,19]. The e-tongue can discriminate between the characteristics of complex mixtures based on the combination of signals from specific and non-specific sensors. For example, Astree II is equipped with three specific and four non-specific sensors. The three specific sensors—AHS, CTS, and NMS—represent sourness, saltness, and umami, respectively, whereas PKS, CPS, ANS, and SCS are general-purpose sensors [20]. The soluble chemical composition of wild vegetables may be altered by pre-drying and drying. The e-tongue can discriminate between the taste properties of the soluble mixture; therefore, it can be applied to assess the effects of pre-drying and drying procedures on the taste variation of dried edible wild vegetables.

Drying is an effective method for extending the shelf life of vegetables. However, the drying process can lead to alterations in texture, color, and taste that are closely linked to the overall quality of the dried produce. It is worth noting that pretreatment methods have the potential to mitigate the drawbacks associated with the drying process, and it is conceivable that the taste of dried vegetables could be influenced by the specific pretreatment employed. Therefore, this study aimed to investigate the effects of NaCl pretreatment prior to using the MOTA technique on the color characteristics and taste of dried *C. setidens* with several health-promoting compounds. Finally, it was discussed whether the taste properties could be reflected by the color characteristics of dried and rehydrated *C. setidens* and whether health-promoting compounds are associated with the taste of *C. setidens*.

## 2. Results

### 2.1. Color Characteristics of C. setidens Processed by the MOTA Technique

**Figure 1:**
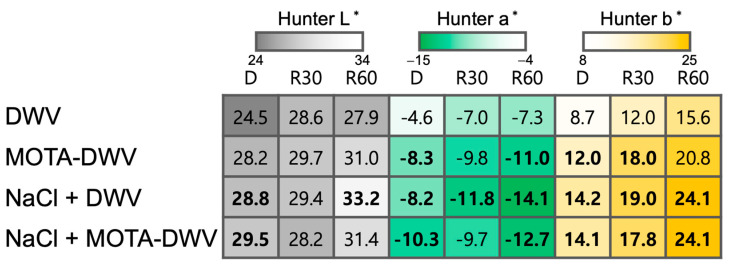
Heat map of Hunter values between treatments. The bold value in the heatmap indicates a significant difference between ‘no treatment’ and other treatments according to a one-way ANOVA with Tukey’s honestly significant difference test (*p* < 0.05; *n* = 6–7). DWV: dried wild vegetable, MOTA-DWV: MOTA-processed dried wild vegetable, NaCl + DWV: dried wild vegetable with NaCl pretreatment, NaCl + MOTA-DWV: MOTA-processed dried wild vegetable with NaCl pretreatment.

Color characteristics and appearance are important quality aspects that determine the preference for DWVs by consumers, as visible color changes occur during the drying process of wild vegetables. To evaluate the effect of NaCl pretreatment followed by using the MOTA technique on the color characteristics of processed *C. setidens*, changes in the visible color of dried and rehydrated *C. setidens* were examined. Dried (D) and rehydrated (R30 (rehydration for 30 min) and R60 (rehydration for 60 min)) MOTA-DWV showed an increase in Hunter L* compared to DWV, but the difference was not significant (Figure 1). However, the NaCl pretreatment significantly raised the Hunter L* values in both the D and rehydrated *C. setidens* (NaCl + DWV). In addition, NaCl + MOTA-DWV showed a significant elevation of the Hunter L* value in dried *C. setidens* (Figure 1), despite no significant elevation of the rehydrated *C. setidens*. These results indicate that visible lightness was improved by NaCl pretreatment. All treatments significantly influenced the Hunter a* and Hunter b* values of dried and rehydrated *C. setidens*. There was an indication of greater greenness and yellowness in dried and rehydrated *C. setidens* processed using the MOTA technique, regardless of NaCl pretreatment, compared to that of DWV. The rehydration rate of MOTA-DWV was also higher than that of DWV (Figure S1 in supplementary materials). Specifically, NaCl + DWV showed a larger improvement in the rehydration rate compared to that of DWV alone, indicating that NaCl has the potential to improve the quality of dried *C. setidens*.

### 2.2. Electronic Tongue Analysis

**Figure 2:**
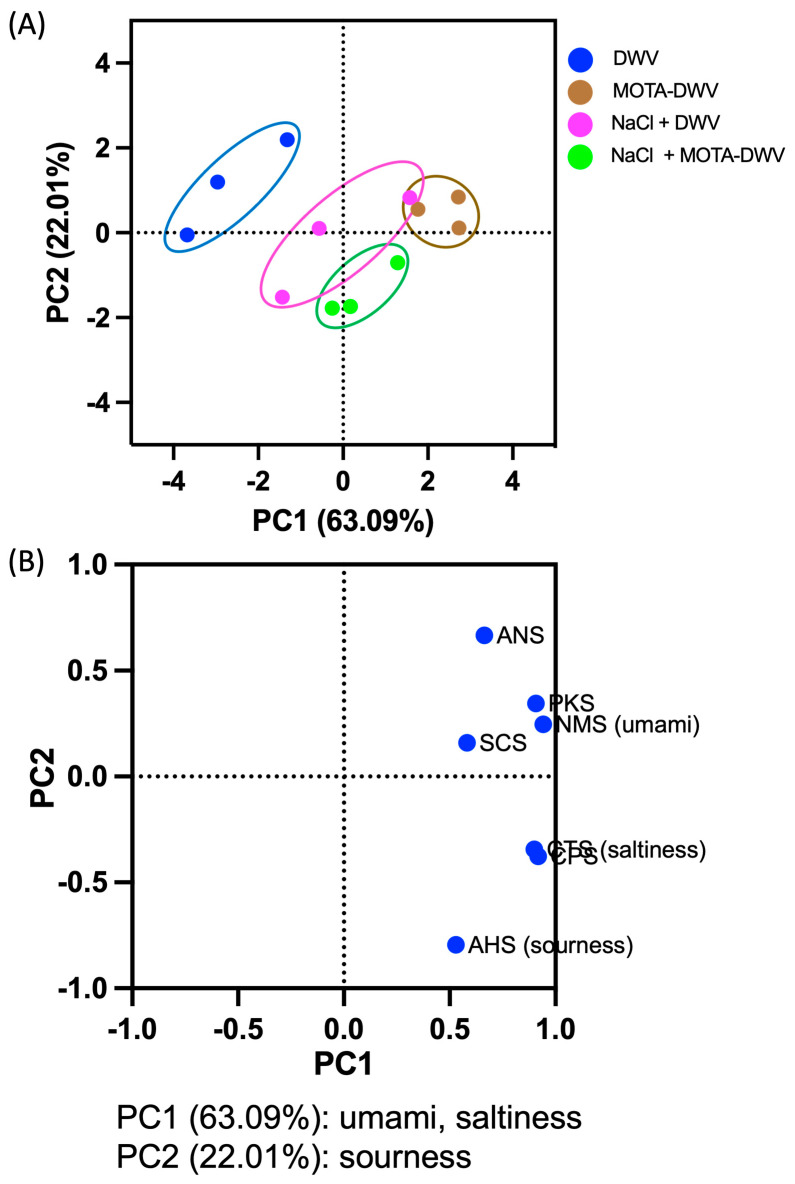
Principal component analysis (PCA) shows relationships between differential signal intensities of the electronic tongue (Astree II) equipped with seven potentiometric sensors. Dried *C. setidens* was infused for 60 min in boiling water, and the infused water was used to measure the taste with the electronic tongue. (**A**) PCA score plot. (**B**) Loading plot. DWV: dried wild vegetable, MOTA-DWV: MOTA-processed dried wild vegetable, NaCl + DWV: dried wild vegetable with NaCl pretreatment, NaCl + MOTA-DWV: MOTA-processed dried wild vegetable with NaCl pretreatment.

To examine the differences in the taste of water-soluble components in *C. setidens* processed using the MOTA technique, *C. setidens*-infused water was analyzed using an Astree II. A data matrix consisting of 12 rows (four samples × three batches) and seven columns (seven sensors) was used to perform PCA. The PCA revealed that the variance contribution rates of PC1 and PC2 were 63.09% and 22.01%, respectively, and the cumulative contribution rate was 85.1% (Figure 2A), indicating that the first two PCs were sufficient to explain the total variance of the datasets. The DWV was separated on the left plane in the PC1 direction and the MOTA-DWV was positioned on the right plane. NaCl + DWV and NaCl + MOTA-DWV were positioned in the middle of the PC1 direction, which was closer to the MOTA-DWV than DWV, indicating that treatment with thermal air contributed to changes in the taste of the water-soluble component of the *C. setidens* and that NaCl pretreatment influenced the final taste of the soluble components of the *C. setidens*. In contrast, none of the groups were clearly discriminated by PC2. To further investigate the major contributors to the principal components, the sensor loadings of PC1 and PC2 were compared. The most significant sensors in PC1 were NMS (umami), CTS (saltiness), CPS, and PKS (Figure 2B). Otherwise, AHS (sourness) contributed significantly to PC2. These data suggest that each process has a distinct influence on specific tastes, such as umami and saltiness in *C. setidens*.

**Figure 3:**
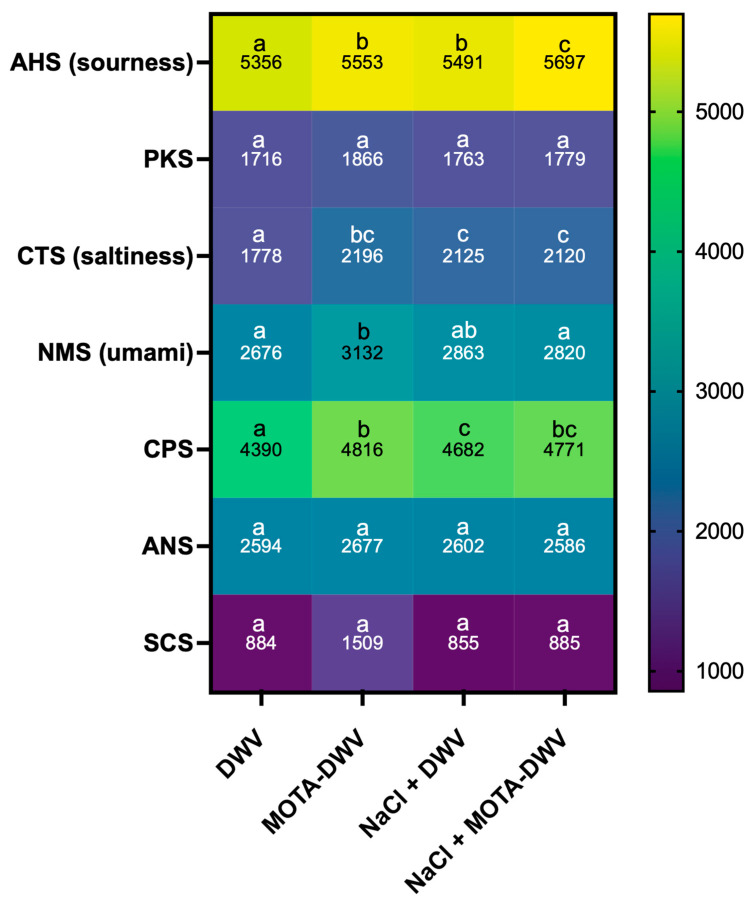
Heat map showing means of signal intensity of electronic tongue (Astree II) between treatments. Different letters indicate a significant difference between treatments according to a one-way ANOVA with Tukey’s honestly significant difference test (*p* < 0.05; *n* = 3). DWV: dried wild vegetable, MOTA-DWV: MOTA-processed dried wild vegetable, NaCl + DWV: dried wild vegetable with NaCl pretreatment, NaCl + MOTA-DWV: MOTA-processed dried wild vegetable with NaCl pretreatment.

**Figure 4:**
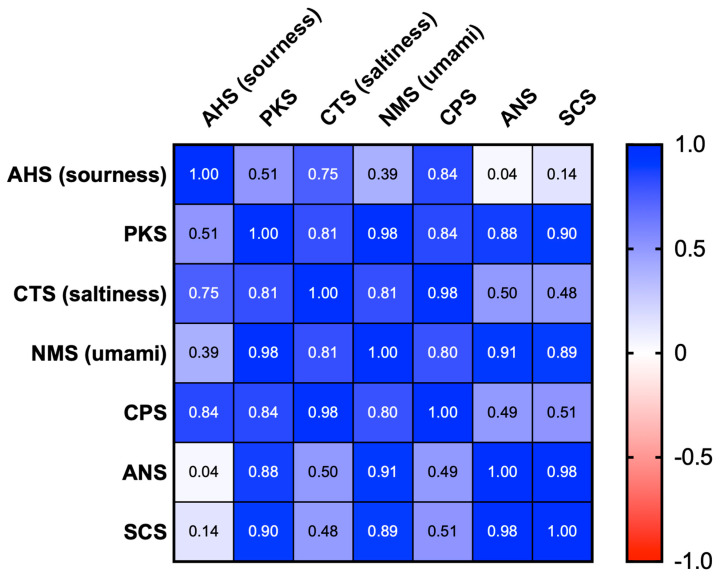
Correlation matrix representing pair-wise Pearson correlation coefficient (values in boxes) matrix between the values of each sensor of the electronic tongue. The color scale depicts the degree of correlation.

The response signal values were compared between treatments (Figure 3). A significant difference between the treatments was observed in the values of AHS (sourness) and CTS (saltiness), with NaCl + MOTA-DWV showing higher values than that of the DWV. In the CPS, all treatments showed significantly higher values than that of the DWV. However, a significant difference in the NMS (umami) value was observed only for the MOTA-DWV. The PKS, ANS, and SCS values were comparable between the treatments. These data suggest that the content of specific soluble components associated with the taste of *C. setidens* varied according to the MOTA technique. Pearson correlation coefficient analysis was used to assess the correlation between the specific taste sensors (AHS, CTS, and NMS) and non-specific sensors (PKS, CPS, ANS, and SCS). All specific taste sensors showed a positive correlation with non-specific sensors (Figure 4). AHS (sourness), CTS (saltiness), and NMS (umami) were highly positively correlated with CPS, with significant differences between the treatments. However, NMS also showed a strong correlation with PKS, ANS, and SCS—the values of which did not exhibit significant differences between treatments. These data suggest that umami was a weaker taste than sourness and saltiness in *C. setidens*-infused water.

### 2.3. Relationship between Color Characteristics and Taste Sensor Values of e-Tongue

**Figure 5:**
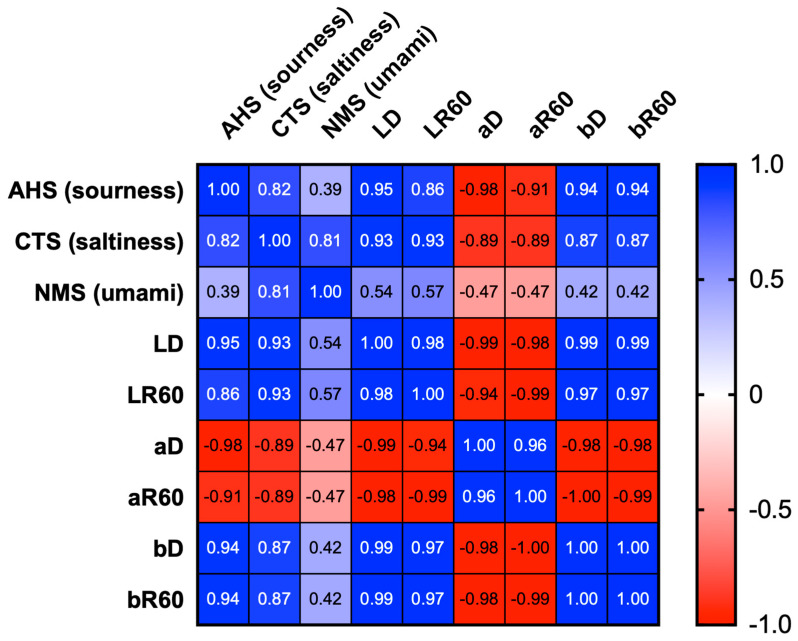
Correlation matrix representing pair-wise Pearson correlation coefficient (values in boxes) matrix between the values of each sensor of the electronic tongue and Hunter L*, a*, and b*. The color scale depicts the degree of correlation.

The color characteristics of fruits and vegetables are closely related to postharvest changes in quality, which can affect their final taste. To evaluate the relationship between color characteristics and the taste of *C. setidens*, a Pearson correlation coefficient test was conducted. The AHS (sourness) and CTS (saltiness) of the infused water with dried *C. setidens* were positively correlated with the value of both the hunter L* and hunter b* regardless of the sample conditions, i.e., either dried or rehydrated *C. setidens* (Figure 5). Moreover, Hunter b* showed a strong negative correlation with the taste sensors. In contrast, NMS (umami) was not significantly correlated with color characteristics. These data suggest that some color characteristics of processed *C. setidens* were associated with specific tastes, such as ‘sourness’ and ‘saltiness’, and it is inferred that the specific tastes could be reflected by the content of the secondary metabolites after processing.

### 2.4. Effect of MOTA Technique on Total Phenolics, Chlorophylls, Carotenoids, and Flavonoids

**Figure 6:**
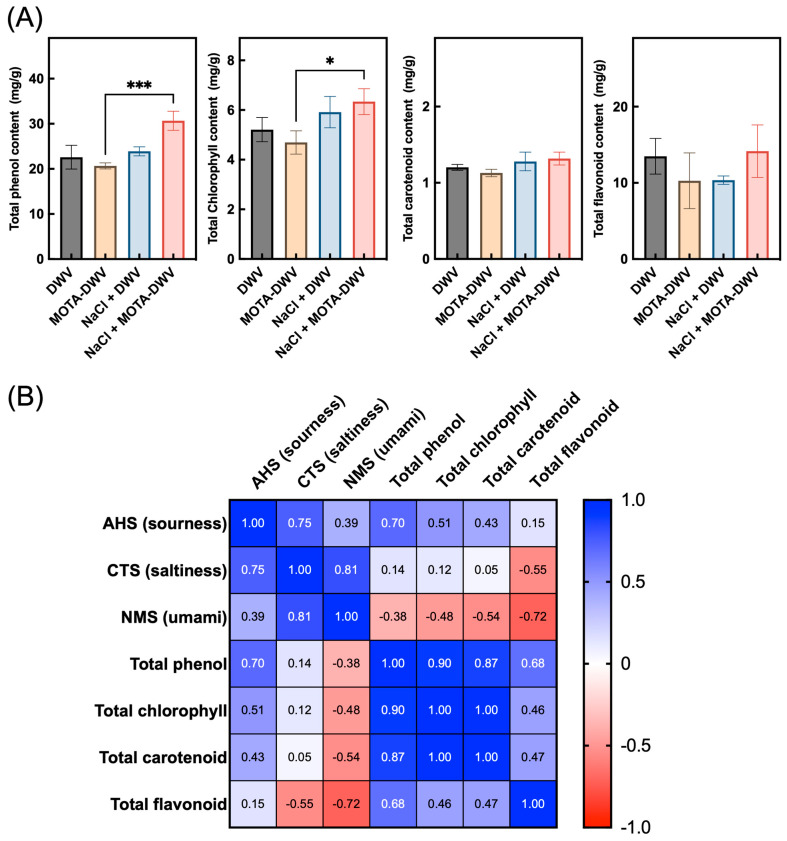
Metabolite content and correlation matrix between the taste and metabolites of processed *C. setidens*. (**A**) Total phenol, total chlorophyll, total carotenoid, and total flavonoid contents. Asterisks indicate significant differences between treatments according to a one-way ANOVA with Tukey’s honestly significant difference test (* *p* < 0.05, *** *p* < 0.001; *n* = 3). (**B**) Correlation matrix representing pair-wise Pearson’s correlation coefficient (values in boxes) matrix between the values of each sensor of electronic tongue and metabolites. The color scale depicts the degree of correlation. DWV: dried wild vegetable, MOTA-DWV: MOTA-processed dried wild vegetable, NaCl + DWV: dried wild vegetable with NaCl pretreatment, NaCl + MOTA-DWV: MOTA-processed dried wild vegetable with NaCl pretreatment.

Plant phenolics, chlorophylls, carotenoids, and flavonoids influence the color, texture, and taste of plant foods [21,22]. Therefore, the effect of the MOTA technique with NaCl pretreatment was examined on the content of secondary metabolites in processed *C. setidens*. A higher total phenolic content was observed in the NaCl + MOTA-DWV than that in the MOTA-DWV (Figure 6A). In addition, NaCl pretreatment had a positive effect on the high chlorophyll content, either without or with the MOTA technique. In contrast, the levels of total carotenoids and flavonoids were not significantly affected by the MOTA technique. These data suggest that the MOTA technique with NaCl pretreatment contributed to the retention of secondary metabolites in processed *C. setidens*. To evaluate the relationship between these metabolites and the specific taste of *C. setidens*, Pearson correlation coefficient analysis was performed between these parameters. There was a highly positive value between secondary metabolites, except for the total flavonoid content (Figure 6B). Likewise, a relatively high positive value was observed between the AHS (sourness) and total phenol content, and the value of the NMS (umami) sensor showed a negative correlation with total flavonoid content, suggesting that the taste of processed *C. setidens* may be attributed to the content of secondary metabolites.

## 3. Discussion

The step of dipping and blanching in an NaCl solution, prior to drying, has been widely applied to improve the color characteristics of dried fruits and vegetables. Tan et al. [23] reported that potato slices treated with NaCl solution underwent less of a color change than untreated potato slices during convective drying combined with infrared heating. Dipping mango slices in NaCl solution combined with citric acid, ascorbic acid, and CaCl_2_—which are enzyme inhibitors associated with browning—followed by hot air drying, contributed to maintaining the color characteristics of the fresh fruit [24]. *Aster scaber* and *C. setidens* blanched with NaCl solution showed improved brightness after drying compared to those blanched with only water [25,26]. NaCl treatment also has an effect on improving the rehydration rate as well as color changes due to its osmotic influence in the drying process of vegetables [27]. Moreover, NaCl plays a crucial role in the dipping and blanching processes as an enzymatic browning inhibitor during the drying process of fresh fruits and vegetables. Furthermore, it was evident that the activity of peroxidase and polyphenol oxidase was lower in dried fruits and wild vegetables treated with NaCl or NaCl-containing color protective solution than in those treated with only water [25,28]. In the present study, blanching *C. setidens* with NaCl resulted in higher Hunter L* and lower Hunter a* values in dried *C. setidens* compared to those of the control sample, regardless of the MOTA technique used (Figure 1). It is possible that blanching with NaCl contributed to the inhibition of the enzymatic color changes of *C. setidens* during the drying process to some extent. Moreover, the MOTA technique combined with blanching in NaCl solution resulted in greater greenness in *C. setidens* than blanching with NaCl alone, indicating that the combination of the MOTA technique and blanching with NaCl solution was a more efficient method of improving the color characteristics of dried *C. setidens*, although there was no significant difference.

Plant phenolics are secondary metabolites and play an important role in developing the color, appearance, flavor, and taste of fruits and vegetables [29,30]. Phenol-derived secondary metabolites, including flavonoid and anthocyanins, are known not only as antioxidants but also as plant pigments that affect the color characteristics of fruits and vegetables [31]. In the fresh *C. setidens*, green was the major color, which meant that chlorophyll was employed to develop the color of the leaves of the *C. setidens*. In a previous study, the color of *C. setidens* processed by the MOTA technique was closer to green than that of dried *C. setidens* [11]. However, the dried *C. setidens* processed by the MOTA technique contained a similar chlorophyll content compared to that with no treatment in the present study (Figure 6), although the Hunter a* value—a minor value representing greenness—was significantly lower than that of untreated *C. setidens* (Figure 1). In contrast, *C. setidens* contained a high content of phenolic compounds compared to the total chlorophyll content (approximately four-fold, Figure 6), and the flavonoids constituted a large portion of phenolics (approximately 50%). Based on these results, it can be inferred that the interaction between the pigments and their content and portions, such as a high content of phenolics in leaf tissue, may determine the final color characteristics of dried or MOTA-processed *C. setidens*. Additionally, the inhibition of phenolic oxidation induced by polyphenol oxidase appears to be another contributing factor in defining these color characteristics.

Phenolic compounds in plants influence the taste of fruit- and vegetable-based foods. Although the taste of phenolics is known to be associated with bitterness and astringency, when phenolic extracts from unripe grapes are added to food models, it allows for changes in taste, such as sourness, saltiness, bitterness, and astringency [32]. Moreover, the addition of phenols to plant-based food models increases their bitterness, sourness, astringency, and pungency [33]. Phenol-rich plant-based foods exhibit specific taste properties such as bitterness and sourness, owing to the presence of polyphenols, isoflavones, and other natural compounds [34,35]. Therefore, phenolic compounds in plant-based foods could affect various taste attributes besides bitterness and astringency. In this study, there was a relatively high positive correlation between the total phenol content and sourness (Figure 6B). Furthermore, NaCl + MOTA-DWV contained the highest phenol content and showed a higher value for the AHS sensor sourness (Figure 3 and Figure 6A) than that of the other samples. These results support those of previous studies showing that phenolic compounds affect the sourness of plant-based foods. Therefore, use of the MOTA technique with NaCl pretreatment could be a new technique to cause ‘sourness’ in wild vegetables by retaining some secondary metabolites without the use of additives.

## 4. Materials and Methods

### 4.1. Plant Material, MOTA Treatment, and Rehydration

Edible wild vegetables (*C. setidens*) were grown in a cultivation field at the National Institute of Forest Science, Suwon, Gyeonggi-do, South Korea and harvested at the commercial maturity stage. The harvested *C. setidens* was washed with tap water thoroughly and then blanched with tap water only or 5% NaCl solution at 90 °C for 3 min. After blanching, the wild vegetables were immediately cooled in cold water and transferred to a spin dryer to remove the residual water. For MOTA processing, each sample was sprayed with 0.2% oil and thermally treated using an air-fryer (DAP-I14DAB; Zhongshan Aouball Electric Appliances Co., Ltd., Zhongshan, China) at 90 °C for 10 min. Finally, the samples were dried using an agricultural dryer (UDS-2511F; KD Navien, Seoul, Republic of Korea) at 45 °C for 12 h. Wild vegetables, dried immediately after blanching, were used as a control treatment. To perform the rehydration process, dried *C. setidens* (300 g dry weight of each DWV, MOTA-DWV, NaCl + DWV, and NaCl + MOTA-DWV) was weighed and soaked in boiling water for specified time intervals (30 and 60 min, respectively). Next, the rehydrated wild vegetables were transferred to a bowl filled with cold water and cooled. The rehydrated wild vegetables were placed in a sieve with overlapped tissue paper to remove residual water.

### 4.2. Color Measurement

The color of the dried and rehydrated *C. setidens* was measured using a Minolta Chroma Meter (Model CR-400; Konica Minolta Optics Inc., Osaka, Japan) at three equidistant points for each wild vegetable. Three samples of wild vegetables were randomly selected for color measurements. To describe the color characteristics between treatments, values were calculated using the Hunter scale, where each value represents L* (lightness), a* (greenness < redness), and b* (blueness < yellowness).

### 4.3. Electronic Tongue Analysis

To analyze the taste of the water-soluble components, dried wild vegetables (DWVs) were infused in boiling water for 60 min, and then the infused water was diluted 100-fold with distilled water. The diluted sample solution was used to measure the taste of the wild vegetables using an electronic tongue (Astree II; Alpha MOS, Toulouse, France) equipped with seven potentiometric sensors. Before sample measurements were conducted, conditioning, calibration, and diagnostics were performed using a standard sample provided by Alpha MOS. Then, each sample (100 mL) was measured for 120 s, and the average values of the last 15–20 s were used to represent the values for each sensor. Three biological replicates were used to calculate the final taste values of the *C. setidens*.

### 4.4. Measurement of Total Phenol Content

DWVs were ground using a mortar and pestle, and the fine powder (0.1 g) was homogenized with acidic MeOH (MeOH: concentrated HCl = 99:1; *v*/*v*) to extract the phenolic compounds. The homogenized mixture was centrifuged at 3000 rpm for 20 min at 10 °C. The total phenolic content of the supernatant was measured using the Folin–Ciocalteu reagent method [36]. The extract (0.1 mL), MeOH (0.1 mL), and the Folin–Ciocalteu reagent (0.1 mL) were mixed in a new tube, and incubated for 6 min in the dark at room temperature. Next, 20% Na_2_CO_3_ (0.7 mL) was added to the tube. The mixture was then vortexed and incubated for 60 min in the dark at room temperature. Finally, the mixture was centrifuged at 13,500 rpm for 3 min at 4 °C. The absorbance of the supernatant was measured at 735 nm using a spectrophotometer (Epoch 2; Agilent Technologies, Santa Clara, CA, USA). A gallic acid standard (Sigma-Aldrich, St. Louis, MO, USA) curve was used to calculate the total phenolic content.

### 4.5. Measurement of Total Chlorophyll, Total Carotenoid, and Total Flavonoid Contents

DWVs were ground using a mortar and pestle, and the fine powder (0.1 g) was homogenized with 80% acetone to extract the pigments. The homogenized mixture was filtered through a filter paper, and the pigment content of the filtered extract was measured. The absorbances at 662, 645, and 470 nm were measured using a spectrophotometer (Epoch 2) to calculate the total chlorophyll and total carotenoid content, according to the methods of Arnon [37] and Win et al. [38]. Total flavonoid content was measured using a previously described method [39]. The extract (1 mL), distilled water (4 mL), and 5% NaNO_2_ (0.3 mL) were mixed in fresh tubes and incubated for 5 min at room temperature. Further, 10% AlCl_3_ (0.3 mL) was then added to the tubes. The tubes were vortexed and allowed to stand for 6 min at room temperature. Finally, 1 M NaOH (2.4 mL) and distilled water (2.4 mL) were added to the tubes and mixed by vortexing. The absorbance of the final solution was measured at 510 nm using a spectrophotometer (Epoch 2). The total flavonoid content was quantified from the absorbance based on a calibration curve that was constructed using catechin (Sigma-Aldrich) standards.

### 4.6. Statistical Analysis

All data are presented as mean ± standard deviation (SD). Student’s *t*-test and one-way ANOVA with Tukey’s honestly significant difference test were used to assess the statistical significance of differences between groups using GraphPad Prism 9. Principal component analysis (PCA) and Pearson correlation coefficient analysis were also performed using GraphPad Prism 9.

## 5. Conclusions

In recent years, the demand for wild vegetables as food ingredients has gradually increased, owing to the potential health benefits from their antioxidant compounds. Most wild vegetables are subjected to drying, which is an inevitable process that prolongs their distribution and storage periods and maintains marketability, as fresh-cut wild vegetables have a short shelf life. However, inadequate drying processes result in nutritional and visible quality losses. Therefore, it is important to develop an optimal drying process for wild vegetables. In a previous study, a new drying technique was developed: the MOTA technique—the main function of which was to use an oil spray, followed by heating in thermal air for a short time before the drying process. This resulted in an improved rehydration rate and color characteristics and shortened the rehydration time after processing. The most obvious finding of this study was that NaCl pretreatment influenced the final color characteristics and taste of MOTA-processed *C. setidens* and that color characteristics were associated with the taste of dried *C. setidens*, which was affected by some health-promoting compounds. Based on these results, it is proposed that the taste of DWVs in the market can be predicted based on their color characteristics. This could help consumers to choose from the wild vegetables available in the marketplace based on their preferences. To the best of our knowledge, this is the first report demonstrating that the color characteristics of dried *C. setidens* are associated with the taste of wild vegetables.

## Data Availability

The data that support the findings of this study are available from the corresponding author upon reasonable request.

## Supplementary Materials

The following supporting information can be downloaded at: https://www.mdpi.com/article/10.3390/plants12183193/s1, Figure S1. Rehydration rate of dried *C. setidens* is processed by MOTA technique with NaCl.
