# Supplementary material for: Effect of NaCl Pretreatment on the Relationship between the Color Characteristics and Taste of Cirsium setidens Processed Using a Micro-Oil-Sprayed Thermal Air Technique
Source: Plants (Basel). 2023 Sep 6;12(18):3193. doi: 10.3390/plants12183193 (PMC10535967; doi:10.3390/plants12183193)
Supplement: Supplementary file 1 [file plants-12-03193-s001.zip › plants-2555382-supplementary.pdf]

SUPPLEMENTARY MATERIAL

TITLE: Effect of NaCl pretreatment on the relationship between the color characteristics and taste of *Cirsium setidens* processed using a micro-oil-sprayed thermal air technique

AUTHORS

Yonghyun Kim[1], Uk Lee[1], and Hyun Ji Eo[1]*

AUTHOR AFFILIATION

[1]Special Forest Resources Division, National Institute of Forest Science, Gwonseon-gu, Suwon 16631, Republic of Korea

***CORRESPONDING AUTHOR**

eohyunji1030@korea.kr

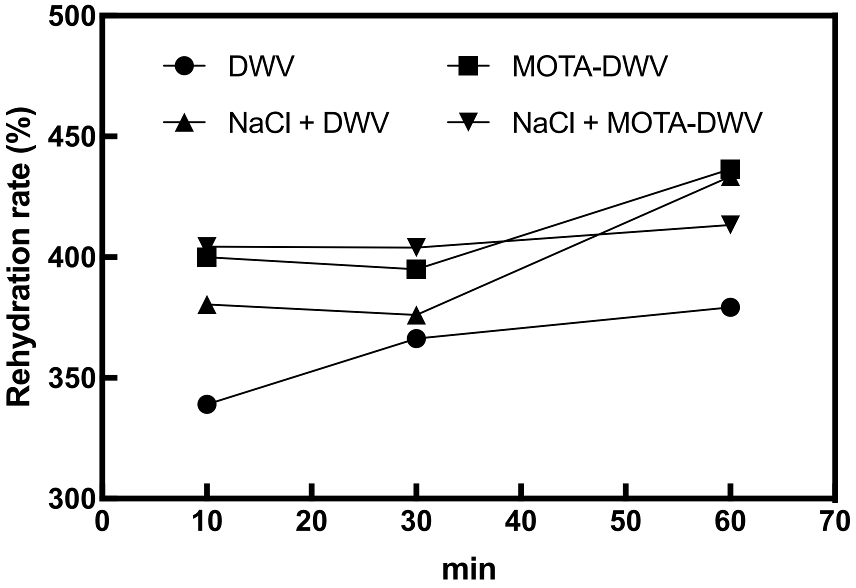

Figure S1. Rehydration rate of dried *C. setidens* is processed by MOTA technique with NaCl. Dried *C. setidens* is soaked in boiling water for 10, 30, and 60 min. DWV: dried wild vegetable, MOTA-DWV: MOTA-processed dried wild vegetable, NaCl + DWV: dried wild vegetable with NaCl pretreatment, NaCl + MOTA-DWV: MOTA-processed dried wild vegetable with NaCl pretreatment.
